# National Vaccine Establishment

**Published:** 1810-12

**Authors:** 


### *NATIONAL VACCINE ESTABLISHMENT.*

***Ordered by the Board, that the following Description of the Vaccine Vesicle, and Instructions relative to Vaccination, which have been presented by the Director, be strictly observed by the Vaccinating Surgeons.***

WHEN Vaccination succeeds, a small red spot is observable on the third day, the day the operation is performed being reckoned reckoned the first. If the Spot is touched, an elevation is felt; and if examined with a magnifying glass the little tumor appears surrounded by a very slight efflorescence.

The Spot gradually enlarges; and between the third and sixth day, a circular Vesicle appears. The edge of the Vaccine Vesicle is elevated, the centre depressed. The colour is at first of a light pink, sometimes of a bluish tint; and changes by degrees to a pearl colour. The centre is somewhat darker than the other parts.

The Vesicle is hard to the touch.

In its internal structure, it is cellular; the cells being filled with transparent lymph.

The Vesicle commonly augments till the tenth or eleventh day.

In the early stages, there is usually round the base an inflamed ring; or this takes place on the seventh or eighth day; towards the ninth it spreads rapidly, and near the tenth forms an Areola of about an inch and a half in diameter.

This Areola is of the usual colour of inflamed skin; it is hard, and accompanied with some degree of tumefaction.

It continues out for a day or two, and then begins to fade; sometimes forming two or three concentric circles.

After the Areola is formed, the Vesicle begins to decline; the centre first turns brown, and the whole gradually changes into a hard, smooth scab, of a very dark mahogany colour.—This dry crust usually drops off about the end of the third week, leaving a permanent cicatrix.

*Varieties in the Progress and Appearance of the Vaccine Vesicle, not preventing the Success of Vaccination.*

The first appearance is seldom earlier, but often later than has been described. In some rare instances the Vesicle commences even a fortnight, or three weeks after Vaccination; but if the process is then regular, it is equally efficacious.

When the Vesicle is slightly ruptured before the sixth day, if it resume its proper form, and the process continue quite regular, success is not prevented: nor is it, when the crust of a regular Vesicle is rubbed off in the decline of the disease.

*The Irregular and Imperfect Vesicle and Pustule, which are not to be depended upon.*

In these deviations there is usually a premature itching, irritation, inflammation, vesication, or suppuration. Or the progress of the Vesicle is too rapid, its texture soft, its edge not well defined, its centre elevated, and the contents discoloured or purulent. Or instead of a proper Areola, a premature efflorescence of a dusky purple hue takes place, and the scab is of a light brown or amber colour.

The

The irregular Vesicle or Pustule is more liable to be broken, than the other, both from its more pointed form, and softer texture, and also from its being usually so irritable as to provoke scratching. When broken, or even without this happening, ulceration often ensues.

A Vesicle apparently regular at first, sometimes does not augment to the proper size, but dies away without compleating the regular process. This usually leaves no cicatrix, or one which is almost imperceptible.

When those, or any other considerable deviations from the regular course of the disease take place, no dependence can be placed upon the operation. In such cases Vaccination should be repeated.

*Probable Causes of Irregular Vesicles and Pustules.*

These accidents may be occasioned by matter or lymph being taken from an irregular Vesicle, or Pustule, at any period, or from a regular Vesicle, at too late a period; or by lymph, though originally pure, which has been injured by long keeping, by heat, or otherwise. Or they may be caused by performing the operation with rusty or unclean lancets, or in a rude manner, or by injuring the Vesicle at an early stage, and thereby exciting too much inflammation, or interrupting the regular progress of the disease. Herpetic eruptions, and other cutaneous affections, have also been supposed the cause of these irregularities; and occasionally to prevent the Vaccine lymph having any effect.

*The Methods of taking Vaccine Lymph for Vaccination.*

The lymph of a regular Vesicle is efficacious from the time it is secreted, till the Areola begins to spread. It may therefore commonly be taken till the ninth day; but not after the Areola is formed.

The lymph is to be taken by small superficial punctures made in the Vesicle, with the point of a lancet. Time should be allowed for the liquid to exude, which will form small pellucid drops. When requisite, a very slight pressure may be cautiously applied with the flat surface of the lancet. Great delicacy is requisite in this operation; for if the Vesicle is rudely treated, or too much opened, inflammation and ulceration may ensue.

Lymph intended to be used immediately, or in a few days, may be received on a lancet; but this is an improper instrument for preserving it longer; for the lymph soon rusts the lancet, and it is then liable to be inefficacious or injurious.—Quills and toothpicks succeed; but small bits of ivory shaped like the tooth of a comb, and properly pointed, are the most convenient instruments; and to render them more certain, they should be charged repeatedly.

In

In order to preserve lymph for a long period, the best method is by two bits of square glass. The lymph is to be received on the centre of one of them, by applying it to a punctured Vesicle. When fully charged and dry, it is to be covered with another bit of glass of the same size, and wrapped up in paper or in gold-beater's skin.

In which ever way the lymph is taken, it should be allowed to dry without heat, in the shade, and be kept in a dry and cool place. When inclosed in a letter, if great care is not taken it may be injured by the heat of the melted wax in sealing the packet.

*The Mode of Vaccinating.*

Liquid lymph is better than dry, because it seldomer fails, and the operation is more lightly and quickly performed. Therefore in every instance, where it is practicable, the patient from whom the lymph is to be taken should be present, and the lymph should be transferred from the one to the other.

Vaccination is generally performed in the arm near the insertion of the deltoid muscle; but in order to hide the scar, and in adults who are likely to use the arm much, it may be advisable to vaccinate the outside of the leg, a little above or below the knee.

The skin being stretched, a lancet charged with Vaccine lymph should be held with its flat surface to the skin; and the point insinuated slantingly through the cuticle till it touches the cutis. It should be retained there for a few seconds.

The lancet should be dipped in water and wiped after each operation, even when several successive inoculations are to be performed.

Dry lymph on glass may be moistened with a very little cold, or tepid water, on the point of a lancet, allowing it some time to dissolve, and blending it by a little friction with the lancet. It must not be much diluted, but of a thick consistence; it is to be inserted in the same manner as the recent fluid.

When quills, ivory lancets, or tooth-picks charged with dry lymph are used, the lymph should not be diluted, but a puncture having been first made with a common lancet, the point of the instrument is to be inserted, and held in the puncture half a minute or more, that the lymph may gradually dissolve and remain in the wound. If the part of the instrument which is charged, be afterwards wiped repeatedly upon the edges of the puncture, it will tend still farther to ensure success.

Vaccinated patients must be cautious not to wear tight sleeves, nor nor to injure the Vesicle by pressure, friction, or any other violence; lest considerable inflammation or ulceration should ensue.

One perfect Vaccine Vesicle is sufficient; but for various reasons, it is always prudent to make two or three punctures, especially when the danger of receiving the Small Pox is imminent, the lymph dry, or the patient's residence distant. Besides, greater security is obtained against a chance of failure from the derangement or destruction of one Vesicle by accidental injury, or by the taking of lymph for Vaccination. When two punctures are to be made in one limb, they should be at least two inches asunder, on account of the irritation they may occasion.

*One Vesicle should be always permitted to go through its course without being punctured.*

Lancets for Vaccination should be kept clean and bright.

*Constitutional Symptoms.*

Constitutional Symptoms sometimes occur at a very early period, but more commonly from the seventh to the eleventh day. These are drowsiness, restlessness, a chilliness succeeded by heat, thirst, head-ache, and other marks of febrile affection. Now and then sickness or vomiting takes place, especially in infants.

The Constitutional Symptoms are in general slight and transient.

In a great proportion of cases there is no perceptible indisposition; nevertheless, the person vaccinated is not the less secure from the future infection of the Small Pox, provided the progress of the Vesicle has been regular and complete.

Care should be taken not to confound the symptoms of other diseases with those produced by Vaccine Inoculation.

*Medical Treatment.*

In general no medicine is required in this mild affection; but if the symptoms happen to run a little higher than usual, the same remedies are to be applied, as if they proceeded from any other cause.

No preparatory medicines are necessary before vaccinating, and commonly no cathartics need be given afterwards.

Should the local inflammation exceed the usual bounds, which rarely happens, unless from tight sleeves, pressure or friction, it may soon be checked by the frequent application of compresses of linen dipped in water, in liquor Plumbi Acetatis dilutus, or in a solution of one drachm of Plumbi Superacetas in a pint of water. These are to be applied cold.

If the scab be rubbed off prematurely, and ulceration takes place, cooling and astringent applications may be used; such as as a drop of liquor Plumbi Acetatis which should be allowed to dry on the part, and then be covered with compresses dipped in water, or in either of the preparations of lead abovementioned, and frequently renewed.

When ulceration is deep or extensive, a poultice either of bread and milk, or of bread with any of the preparations of lead, may be applied, as the case seems to require. They must never be applied till they are nearly, or quite cold.

In such foul and obstinate sores as resist the foregoing applications, the Unguentum hydrargyri nitratis, mixed with an equal quantity of Unguentum Cetacei or other similar applications, may sometimes be resorted to with advantage. And at other times these sores may be healed by the Ceratum Plumbi Superacetatis, or the mildest applications.

The irregular Vesicles and Pustules are frequently followed by ulceration at an early period, which is to be treated in the same manner, as if it proceeded from the regular Vesicle.

When the patient has been previously exposed to the infection of Small Pox, this disease will be either superseded or not, according to the time which has elapsed before Vaccination.

Medical Gentlemen in all parts of the Empire may be supplied with Vaccine Lymph, without any expense, from the National Vaccine Establishment.

Applications for Lymph, Letters, and Communications respecting Vaccination, will meet with proper attention:—they should be addressed to Dr. Hervey, Register, Leicester Square; and when from a distance put under a cover, directed to the Right Hon. the Secretary of State for the Home Department.

*Board Room*, 21, *Leicester Square*, 1810.

